# Genotype-by-environment interactions for feed efficiency traits in Nellore cattle based on bi-trait reaction norm models

**DOI:** 10.1186/s12711-023-00867-2

**Published:** 2023-12-14

**Authors:** João B. Silva Neto, Lucio F. M. Mota, Sabrina T. Amorim, Elisa Peripolli, Luiz F. Brito, Claudio U. Magnabosco, Fernando Baldi

**Affiliations:** 1 Department of Animal Science, School of Agricultural and Veterinarian Sciences (FCAV), São Paulo State University (UNESP), Jaboticabal, SP 14884-900 Brazil; 2 School of Animal Sciences, Virginia Polytechnic Institute and State University, Blacksburg, VA 24061 USA; 3 School of Veterinary Medicine and Animal Science, University of São Paulo, Pirassununga, SP 13635-900 Brazil; 4 Department of Animal Sciences, Purdue University, West Lafayette, IN 47907 USA; 5 Embrapa Rice and Beans, GO-462, km12, Santo Antônio de Goiás, GO 75375-000 Brazil

## Abstract

**Background:**

Selecting animals for feed efficiency directly impacts the profitability of the beef cattle industry, which contributes to minimizing the environmental footprint of beef production. Genetic and environmental factors influence animal feed efficiency, leading to phenotypic variability when exposed to different environmental conditions (i.e., temperature and nutritional level). Thus, our aim was to assess potential genotype-by-environment (G × E) interactions for dry matter intake (DMI) and residual feed intake (RFI) in Nellore cattle (*Bos taurus indicus*) based on bi-trait reaction norm models (RN) and evaluate the genetic association between RFI and DMI across different environmental gradient (EG) levels. For this, we used phenotypic information on 12,958 animals (young bulls and heifers) for DMI and RFI recorded during 158 feed efficiency trials.

**Results:**

The heritability estimates for DMI and RFI across EG ranged from 0.26 to 0.54 and from 0.07 to 0.41, respectively. The average genetic correlations (± standard deviation) across EG for DMI and RFI were 0.83 ± 0.19 and 0.81 ± 0.21, respectively, with the lowest genetic correlation estimates observed between extreme EG levels (low vs. high) i.e. 0.22 for RFI and 0.26 for DMI, indicating the presence of G × E interactions. The genetic correlation between RFI and DMI across EG levels decreased as the EG became more favorable and ranged from 0.79 (lowest EG) to 0.52 (highest EG). Based on the estimated breeding values from extreme EG levels (low vs. high), we observed a moderate Spearman correlation of 0.61 (RFI) and 0.55 (DMI) and a selection coincidence of 53.3% and 40.0% for RFI and DMI, respectively.

**Conclusions:**

Our results show evidence of G × E interactions on feed efficiency traits in Nellore cattle, especially in feeding trials with an average daily gain (ADG) that is far from the expected of 1 kg/day, thus increasing reranking of animals.

**Supplementary Information:**

The online version contains supplementary material available at 10.1186/s12711-023-00867-2.

## Background

In recent years, various worldwide beef cattle breeding programs have included feed efficiency-related traits as a selection criterion to increase the profitability of beef production and minimize the industry’s environmental impact. Feeding represents approximately 70% of the total beef cattle production costs [1], and improving individual feed efficiency could potentially influence the profitability and sustainability of beef cattle production systems [2]. Feed efficiency-related traits are controlled by different physiological and biological processes that are associated with feed intake and energy expenditure [3, 4]. In this context, variation in feed composition and availability may result in different weight gain ratios, which could potentially be impacted by genotype-by-environment (G × E) interactions [5].

Both genetic and environmental factors affect individual feed efficiency traits, leading to phenotypic variability in response to exposure to divergent environmental conditions [6–8]. Previous studies indicated that animals that are fed high-energy diets had a significantly improved feed efficiency as compared to those fed low-energy diets [9]. In this context, evaluating the evidence of G × E interactions on economically important traits is essential to selecting breeding animals with progeny showing good performance even under challenging conditions [8, 10, 11].

In spite of several studies that have evaluated feed efficiency traits under the assumption of independent genetic and environmental effects in the models used to estimate genetic parameters [12–15], the effect of G × E interactions on the genetic parameters and breeding values for feed efficiency traits, such as dry matter intake (DMI) and residual feed intake (RFI), across environmental gradients (EG) remain unknown in Nellore cattle (*Bos taurus indicus*) populations. In taurine breeds (*Bos taurus taurus)*, Durunna et al. [9] reported evidence of G × E interactions for RFI and DMI in crossbred steers fed with growing and finishing diets.

G × E interactions occur when a genotype expresses different performances in different environmental or management conditions [6]. G × E interactions can be assessed based on genetic correlations for the same trait measured across environments. Genetic correlations lower than 0.80 have been suggested to indicate a significant effect of G × E interactions on the target traits [7], with potential reductions in selection response [8]. In this context, G × E interactions represent a major challenge for breeding programs, as they are an important source of phenotypic variation in animals raised across different environments. G × E interactions also affect the genetic variance and re-ranking among selection candidates [16, 17].

In Brazil, feed efficiency trials have been carried out on experimental stations and commercial herds across diverse geographical and climatic regions or nutritional strategies [18]. Although an average daily gain (ADG) of approximately 1 kg/day is recommended for feeding trials [5], possible differences in diet composition and environmental conditions during different feeding trials may result in differences in ADG. In this context, animals from herds with a greater selection emphasis on growth traits tend to have a higher genetic merit for ADG, which influences the feeding intake needed to meet their energy requirements and, consequently, the RFI results. Thus, comparisons of the expected estimated breeding values (EBV) between feeding trials conducted under different management conditions, with different diet compositions, and with animals from multiple herds are often challenging [9, 19]. Although most feed efficiency trials follow standard nutritional recommendations for diet formulation, the chemical composition of the diets can lead to divergent nutrient intake [20].

In beef cattle, G × E interactions have been evaluated for several traits based on reaction norm (RN) models and continuous EG levels, including the estimated average performance of contemporary groups (CG) [21–23]. The RN model links the phenotypic variability to an environmental value through the polynomial function, where the polynomial coefficients indicate the expected average EBV of the animal (intercept) and the slope coefficient represents the animal response to environmental changes [24–26]. Evaluating G × E interactions is crucial to optimize the design of breeding programs and enhance the genetic improvement of feed efficiency-related traits measured across environments. Thus, the aim of this study was to assess the level of G × E interactions for DMI and RFI in Nellore cattle based on a bi-trait RN model and evaluate the genetic correlation between DMI and RFI across EG levels.

## Methods

### Field data

The phenotypic information for feed efficiency-related traits was measured on 12,958 Nellore animals (9170 males and 3788 females) and was provided by the National Association of Breeders and Researchers (ANCP, Ribeirão Preto, SP, Brazil; www.ancp.org.br). Animals were recorded during 158 feeding trials and belonged to three commercial herds (Nelore HoRa, Cornélio Procópio, PR; Rancho da Matinha, Uberaba, MG; and AgroNova, Barra do Garças, MT, Brazil) and two research centers (Embrapa Cerrados, Goiânia, GO; and Federal University of Uberlândia, Uberlândia, MG, Brazil). The dataset used includes phenotypic information for ADG, DMI, and RFI, following the procedures for measuring individual feed intake in beef cattle [5]. The datasets are highly connected due to the use of common sires across herds through artificial insemination (AI), with at least five genetic links across the feeding trials, which were evaluated using the AMC program [27]. The animals were raised on pasture-based systems (*Urochloa brizantha cv*). The commercial herds adopt different nutritional practices with some farms providing protein and mineral supplementation, especially during the dry season, while others providing only urea supplementation.

### Phenotypic information

The animals received an ad libitum mixed diet during the feeding trials, allowing refusals from 5 to 10%. The feeding trial was performed in group pens from 2011 to 2017 with animals grouped by sex. Feed intake was recorded automatically based on the GrowSafe (www.vytelle.com) and Intergado (www.intergado.com) feeding systems. The feeding trials comprised at least 21 days for adaptation to the feedlot diet and management and an average of 64.74 ± 29.6 days for the data collection period of DMI and ADG. Animals were weighed without fasting at the beginning and end of the feeding trial and every 14 days during the experimental period. Total mixed ration (TMR) was offered over the years but differed in composition and ingredients. Diets were formulated as described by Mendes et al. [5], based on corn silage and commercial concentrate, with an average of 64% of total digestible nutrients (TDN), 13% of crude protein (CP), 76% of dry matter (DM), and formulated for different weight gains/day. The diets were adjusted based on the percentage of dry matter (%DM) to guarantee 2.17 Mcal/kg for metabolizable energy (ME) and 1.3 MJ/kg for net energy for gain (NE_g_). In addition, samples of roughage, concentrate, and diet refusals were collected to evaluate their chemical composition, such as %DM, which is crucial for evaluating DMI and feed efficiency. Thus, the %DM in the diet was determined from weekly samples of the diets offered and refused.

The DMI was estimated as the feed intake per animal recorded automatically by the GrowSafe or Intergado feeding system with subsequent adjustments for dry matter content and expressed as kg/day. ADG was defined as the slope from the linear regression of body weight (BW) on feeding trial days. Finally, residual feed intake (RFI) was estimated within each contemporary group (CG), which was defined by year and season of the feeding trial, farm, sex (males and females were evaluated in different groups) and management groups, as the difference between the observed and expected feed intake considering each animal’s average metabolic body weight (MBW) and ADG, using the equation proposed by Koch et al. [19] as follows:

$${\text{DMI}}={{\text{b}}}_{0}+{{\text{b}}}_{1}{\text{AGD}}+{{\text{b}}}_{2}{\text{MBW}}+{\text{e}},$$

where ${{\text{b}}}_{0}$ is the intercept, ${{\text{b}}}_{1}$ and ${{\text{b}}}_{2}$ are the linear regression coefficients for ADG and MBW, respectively, and ${\text{e}}$ is the residual effect representing the RFI estimate. The MBW was calculated as:

$${\text{MBW}}={\left[\mathrm{\alpha }+{\text{b}}*\left(\frac{{\text{DFT}}}{2}\right)\right]}^{0.75},$$

where $\mathrm{\alpha }$ is the intercept of the regression equation which represents the body weight at the beginning of the feeding trial test; and ${\text{b}}$ is the linear regression coefficient which represents the ADG; and DFT is the number of days of the feeding trial. The descriptive statistics for DMI and RFI are in Table 1.

**Table 1:** Descriptive statistics for dry matter intake (DMI), residual feed intake (RFI), and average daily liveweight gain (ADG) in Nellore cattle and feeding trials information

Variable	RFI (kg/day)	DMI (kg/day)	ADG (kg/day)
Average	− 0.001	8.496	1.205
Standard deviation	− 0.720	2.063	0.350
Minimum	− 4.931	3.171	− 0.403
Maximum	4.698	18.748	4.335
Feeding trials information			
Number of trials with only males	118		
Number of trials with only females	40		
Animals in the pedigree	23,665		
Sires	802		
Dams	6833		
Sires with progeny records	510		
Dams with progeny records	6349		
Number of contemporary groups	505		

### Reaction norm models

A reaction norm model with two steps [11, 28] was considered in the present study. In the first step, the ADG during the feeding trials was used to define the EG levels, given that the actual ADG shows significant variation from the recommended ADG of 1.0 kg per day [5]. The best linear unbiased estimates (BLUE) solutions of CG for ADG were used to quantify potential differences between the management and the environmental conditions (i.e., nutritional differences) during the feeding trials.

### First step—estimation of the environmental gradient levels

The EG levels describing the environmental condition were based on the BLUE solutions of CG for ADG as they are expected to capture differences in management and environmental factors experienced by the animals during the feeding trials. The CG solutions were obtained considering an animal model via best linear unbiased prediction (BLUP) as follows:

$$\mathbf{y}=\mathbf{X}\mathbf{b}+\mathbf{Z}\mathbf{a}+\mathbf{e},$$

where $\mathbf{y}$ is the vector of phenotypic information for ADG; **b** is the vector of fixed effects of CG and age at feeding trials as a linear covariate, $\mathbf{a}$ is the vector of additive genetic effects assumed to follow $N(\boldsymbol{0},\mathbf{A}{\upsigma}_{{\text{a}}}^{2})$, where ${\upsigma }_{{\text{a}}}^{2}$ is the additive genetic variance for ADG and $\mathbf{A}$ is the pedigree relationship matrix; and $\mathbf{e}$ is the vector of residual effects assumed $N(\boldsymbol{0},\mathbf{I}{\upsigma}_{{\text{e}}}^{2})$, where $\mathbf{I}$ is an identity matrix and ${\upsigma }_{{\text{e}}}^{2}$ is the residual variance. $\mathbf{X}$ and $\mathbf{Z}$ are the incidence matrices related to the systematic and additive genetic effects, respectively. The EG levels were obtained by standardizing the BLUE solutions of CG to a mean of 0 and a standard deviation (SD) of 1.

### Second step—reaction norm model

Genetic parameters for DMI and RFI across the EG levels were estimated based on a bi-trait reaction RN model as follows:

$${\mathbf{y}}_{\mathbf{i}\mathbf{j}}=\mathbf{X}\mathbf{b}+{\sum }_{f=0}^{1}{{\varvec{\upomega}}}_{\mathbf{f}}{\boldsymbol{\Phi}}_{\mathbf{f}}\left({\mathbf{E}\mathbf{G}}_{\mathbf{j}}\right)+{\sum }_{f=0}^{1}{{\varvec{\upalpha}}}_{\mathbf{f}\mathbf{i}}{\boldsymbol{\Phi}}_{\mathbf{f}}\left({\mathbf{E}\mathbf{G}}_{\mathbf{j}}\right)+{\mathbf{e}}_{\mathbf{i}\mathbf{j}},$$

where ${\mathbf{y}}_{\mathbf{i}\mathbf{j}}$ is the vector of phenotypic records for DMI and RFI of the animal ${\text{i}}$ on EG level ${\text{j}}$, $\mathbf{b}$ is the vector of fixed effect of CG and age of animal as a linear covariate, $\mathbf{X}$ is the incidence matrix, ${{\varvec{\upomega}}}_{\mathbf{f}}$ is the vector of the ${\text{f}}$-th fixed regression coefficient for intercept ($f=0$) or slope ($f=1$) on ${\boldsymbol{\Phi}}\left({\mathbf{E}\mathbf{G}}_{\mathbf{j}}\right)$; ${\boldsymbol{\Phi}}_{\mathbf{f}}\left({\mathbf{E}\mathbf{G}}_{\mathbf{j}}\right)$ is the vector containing the ${\text{f}}$-th linear Legendre orthogonal polynomials corresponding to EG level ${\text{j}}$ (${{\text{EG}}}_{{\text{j}}})$, ${{\varvec{\upalpha}}}_{\mathbf{f}\mathbf{i}}$ is the vector of the random regression coefficients for the additive genetic effects of the intercept ($f=0$) or slope ($f=1$) corresponding to animal ${\text{i}}$ on EG level ${\text{j}}$, and ${\mathbf{e}}_{\mathbf{i}\mathbf{j}}$ is a vector of random residual effects. The bi-trait RN was evaluated considering the residual variances as homogeneous or heterogeneous across EG levels. For heterogeneous residual variances, the different classes of residual variance were determined using the K-means clustering approach [29] and the best model with different classes of residual variance was chosen based on the Bayesian information criterion (BIC) [30]. For this we considered 11, nine, seven, six, and five classes of residual variance (Table 2). In addition, a quadratic Legendre orthogonal polynomial was tested for the best linear model (Table 3).

**Table 2:** Group of environment gradient (EG) considered for each class of residual variance

Class	EG1 (− 1.5)	EG2 (− 1.2)	EG3 (− 0.9)	EG4 (− 0.6)	EG5 (− 0.3)	EG6 (0.0)	EG7 (0.3)	EG8 (0.6)	EG9 (0.9)	EG10 (1.2)	EG11 (1.5)
Eleven	CL1	CL2	CL3	CL4	CL5	CL6	CL7	CL8	CL9	CL10	CL11
Nine	CL1	CL1	CL2	CL2	CL3	CL4	CL5	CL6	CL7	CL8	CL9
Seven	CL1	CL2	CL2	CL2	CL3	CL3	CL3	CL4	CL5	CL6	CL7
Six	CL1	CL2	CL2	CL2	CL3	CL3	CL3	CL4	CL5	CL6	CL6
Five	CL1	CL2	CL2	CL2	CL3	CL3	CL3	CL4	CL5	CL5	CL5

CL: class of residual variance

**Table 3:** Comparison of the reaction norm models according to the log-likelihood function (LogL) and Schwarz-Bayesian information criterion (BIC) for dry matter intake (DMI) and residual feed intake (RFI) in Nellore cattle

Model	PO	CRV	LogL	BIC	NP
Lin_hom	2	1	2988.3	3054.1	13
Lin_het_11	2	11	2805.9	3023.5	43
Lin_het_9	2	9	2811.3	2998.6	37
Lin_het_7	2	7	2830.0	2986.9	31
*Lin_het_6*	*2*	*6*	*2819.0*	*2960.7*	*28*
Lin_het_5	2	5	2853.2	2979.7	25
Qua_het_6	3	6	2823.6	3021.0	39

Italic: model used; CRV: Residual variance classes; Lin_hom: linear model with homogeneous residual variance; Lin_het_11: linear model with eleven classes of residual variance; Lin_het_9: linear model with nine classes of residual variance; Lin_het_7: linear model with seven classes of residual variance; Lin_het_6: grouping the last two classes of residual variance; lin_het_5: model lin_het_7 grouping the last three classes of residual variance; Qua_het_6: quadratic model with six classes of residual variance

The genetic variance (${\widehat{\upsigma}}_{{\text{a}}{{\text{EG}}}_{{\text{j}}}}^{2}$) and heritability (${\widehat{\text{h}}}_{{{\text{EG}}}_{{\text{j}}}}^{2}$) estimates for DMI and RFI across the EG levels were calculated based on the following equations: ${\widehat{\upsigma}}_{{\text{a}}{{\text{EG}}}_{{\text{j}}}}^{2}\text{=}{\boldsymbol{\Phi}}_{\mathbf{f}}{\mathbf{K}}_{\mathbf{a}\mathbf{b}}{\boldsymbol{\Phi}}_{\mathbf{f}}{\prime}$; where ${\mathbf{K}}_{\mathbf{a}\mathbf{b}}$ is the matrix of estimated (co)variances pertaining to the random regression coefficients for the additive genetic effects of the intercept and slope. The heritability (${\widehat{\text{h}}}_{{{\text{EG}}}_{{\text{j}}}}^{2}$) for each EG level was determined as follows: ${\widehat{\text{h}}}_{{{\text{EG}}}_{{\text{j}}}}^{2}\text{=}\frac{{\widehat{\upsigma}}_{{\text{a}}{{\text{EG}}}_{{\text{j}}}}^{2}}{{\widehat{\upsigma}}_{{\text{a}}{{\text{EG}}}_{{\text{j}}}}^{2}\text{+}{\widehat{\upsigma}}_{{\text{e}}{{\text{EG}}}_{{\text{j}}}}^{2}}$; ${\widehat{\upsigma}}_{{\text{a}}{{\text{EG}}}_{{\text{j}}}}^{2}$ is the additive genetic variance and ${\widehat{\upsigma}}_{{\text{e}}{{\text{EG}}}_{{\text{j}}}}^{2}$ is the residual variance considering heterogeneous variance for EG level ${\text{j}}$. The genetic correlation across EG levels (${{\text{r}}}_{{\text{EGj}},\mathrm{EGj^{\prime}}}$) was determined as follows: ${{\text{r}}}_{{\text{EGj}},\mathrm{ EGj{\prime}}}={\upsigma}_{{\text{EGj}},\mathrm{EGj^{\prime}}}/\sqrt{{\widehat{\upsigma}}_{{\text{a}}{{\text{EG}}}_{{\text{j}}}}^{2}*{\widehat{\upsigma}}_{{\text{a}}{{\text{EG}}}_{\mathrm{j^{\prime}}}}^{2}}$, where ${\upsigma}_{{\text{EGj}},\mathrm{EGj^{\prime}}}$ represents the covariance between EG level ${\text{j}}$ and EG level $\mathrm{j^{\prime}}$, which are estimated in the same way as the additive genetic variance for each EG level.

The EBV for animal i at each EG level were obtained using the following equation: ${\widehat{\text{g}}}_{{\text{i}}{{\text{EG}}}_{{\text{j}}}}\text{=}{{\varvec{\upalpha}}}_{\mathbf{f}\mathbf{i}}{\boldsymbol{\Phi}}_{\mathbf{f}}\mathbf{^{\prime}}$; where ${{\varvec{\upalpha}}}_{\mathbf{f}\mathbf{i}}$ is the vector of the additive genetic values for the intercept and slope estimates of animal ${\text{i}}$ and ${\boldsymbol{\Phi}}_{\mathbf{f}}\mathbf{^{\prime}}$ is the transposed vector of the Legendre orthogonal polynomials for each EG level. Pearson’s and Spearman’s rank correlation coefficient for EBV across EG levels were used to assess the reranking of 50 sires that were selected based on the highest EBV values for a medium EG level (EG = 0) and with at least five progenies recorded at low, medium, and high EG levels. To evaluate the sire coincidence between the low, medium, and high EG levels, we selected 15 sires with the highest EBV in these EG levels.

The genetic analyses were performed using the Wombat software [31] based on the average information restricted maximum likelihood (AI-REML) algorithm. The models were compared using the BIC [30], according to the following equation: ${\text{BIC}}=-2{\text{logL}}+{\text{plog}}({\text{N}}-{\text{r}}\left({\text{X}}\right))$, where ${\text{p}}$ is the number of parameters estimated for the model, ${\text{N}}$ is the number of phenotypic records for DMI and RFI, ${\text{r}}\left({\text{X}}\right)$ is the rank of the coefficient matrix of fixed effects in the analyzed model, and ${\text{logL}}$ is the maximum log-likelihood.

### Environmental sensitivity

A plasticity scale was assumed based on the absolute individual value of the slope *(*${f}_{1}$) and standard deviation of the population slope (${\upsigma}_{f1}$). The animals were classified as robust ($\left|{f}_{1}\right|<{\upsigma}_{f1}$), plastic (${\upsigma}_{f1}<\left|{f}_{1}\right|<{2\upsigma}_{f1}$), and highly plastic ($\left|{f}_{1}\right|>{2\upsigma}_{f1}$).

## Results and discussion

### Comparison of reaction norm models

Based on the BIC criteria, on the one hand, a linear model considering heterogeneous residual variances with six classes (Lin_het_6) was the most appropriate model to fit the residual structure of DMI and RFI, as presented in Table 3. On the other hand, a quadratic model considering six classes of residual variance (Qua_het_6) did not improve the model fit of the data. Among the models tested, the model that assumed homogeneous residual variances (Lin_hom) showed the highest (worst) BIC value. Thus, models considering heterogeneous residual variances fitted the data better than those considering a homogeneous residual variance. To select the optimal model, Meyer [32] recommended to balance the classes of residual variance and the amount of data available, especially when the data are irregularly distributed. In this context, Carvalheiro et al. [33], Mota et al. [11] and Carvalho Filho et al. [34], suggested that for the evaluation of G × E interactions for productive and reproductive traits in Nellore cattle, RN models with heterogeneous residual variances provided a better fit to the data than homogeneous residual variances.

### Phenotypic means of RFI, DMI, and ADG across EG levels

The phenotypic means and standard deviation by EG for the traits studied are in Table 4. For DMI and ADG, as the environment became more favorable (or less restricted), the mean values showed an increasing pattern, ranging from 6.255 to 11.498 (kg of DM/day) for DMI and from 0.680 to 1.928 (kg/day) for ADG. Following the recommendations that a diet energy intake that allows an ADG of around 1.0 kg/day during the feeding trials should be provided [5], there was a large variability in ADG across EG (Table 4). The potential physicochemical differences in the ingredients used for the formulation of the diets, which were caused by the vast climatic variation and geographic regions across the Brazilian regions where the trials were conducted, in addition to the greater selection emphasis on body weight for specific farms, might explain the considerable variation observed in the average ADG of the animals evaluated in this study.

**Table 4:** Number of records (N) and descriptive statistics for dry matter intake (DMI), residual feed intake (RFI) and average liveweight gain (ADG) by environmental gradient (EG) in Nellore cattle

EG*	N	DMI (kg DM/day)	RFI (kg DM/day)	ADG (kg/day)
		Mean ± SD		
1 (− 1.50)	254	6.255 ± 1.036	0.00 ± 0.512	0.680 ± 0.191
2 (− 1.20)	1312	6.907 ± 1.027	0.00 ± 0.649	0.851 ± 0.224
3 (− 0.90)	1938	7.379 ± 1.531	0.00 ± 0.652	0.978 ± 0.205
4 (− 0.60)	2494	7.846 ± 1.278	0.00 ± 0.651	1.093 ± 0.208
5 (− 0.30)	2117	8.472 ± 2.027	0.00 ± 0.724	1.201 ± 0.222
6 (0.00)	1646	9.419 ± 2.553	0.00 ± 0.758	1.313 ± 0.236
7 (0.30)	1512	9.677 ± 1.691	0.00 ± 0.726	1.445 ± 0.289
8 (0.60)	834	10.674 ± 1.690	0.00 ± 1.026	1.566 ± 0.298
9 (0.90)	499	10.349 ± 1.578	0.00 ± 0.616	1.698 ± 0.239
10 (1.20)	151	10.353 ± 1.746	0.00 ± 0.840	1.817 ± 0.225
11 (1.50)	201	11.498 ± 0.094	0.00 ± 0.980	1.933 ± 0.292

DM: dry matter

^*^Values in parentheses represent the environmental gradient solution standardized for mean zero and standard deviation of 1 for ADG BLUE (best linear unbiased estimate) solutions

In pigs, differences in feed composition have been reported as a crucial source of environmental variation and G × E interaction [35], with ADG, DMI, and RFI showing sensitivity to these variations in environmental conditions. Furthermore, improving feed efficiency in production systems generally increases environmental sensitivity, whereby differences in dietary energy concentration significantly impact feed efficiency outcomes [36]. Therefore, it is important to monitor and manage the impact of selection across different feeding trials to improve these traits successfully.

### Heritability, phenotypic, and additive genetic variance estimates

**Fig. 1:**
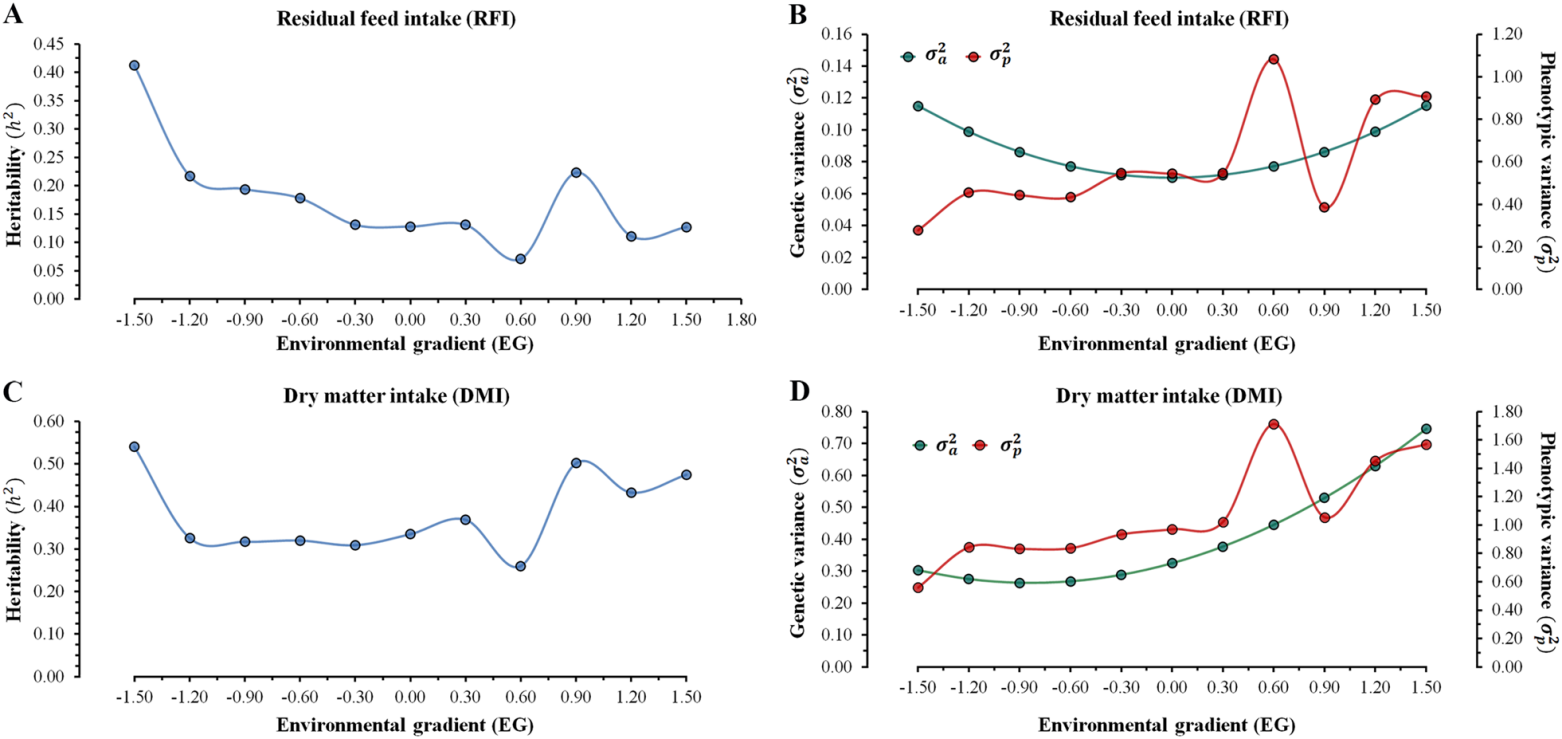
Heritability (${\mathbf{h}}^{2}$) for residual feed intake (RFI) (**a**) and dry matter intake (DMI) (**c**), and additive genetic variance (${{\varvec{\upsigma}}}_{\mathbf{a}}^{2}$) and phenotypic variance (${{\varvec{\upsigma}}}_{\mathbf{p}}^{2}$) estimates for RFI (**b**) and DMI (**d**) across environmental gradients

The heritability estimates obtained for RFI and DMI across EG ranged from 0.07 to 0.41 (Fig. 1a) and from 0.26 to 0.54 (Fig. 1c), respectively. Both RFI and DMI showed similar patterns for heritability estimates across EG, i.e., first decreasing from the lower EG level (− 1.5) until a medium EG level (0.60) and then increasing for higher EG (0.90) levels. Differences in heritability estimates across different EG levels occur due to the effect of G × E interactions leading to changes in genetic and phenotypic variances between EG levels for RFI (Fig. 1b) and DMI (Fig. 1d). Based on these results, it seems that environmental factors might have a greater impact on phenotypic variations than additive genetic effects. This could be due to the enhanced EG level leading to higher ADG or to the possibility of the animal’s genetic potential being masked by the environment.

The heritability estimates obtained for RFI (0.13) and DMI (0.34) for the medium EG level (0.0) were slightly different from those reported in the literature, with models that do not consider G × E interactions. However, the values across EG levels (Fig. 1a and c) corroborate with the expected values observed in the literature. The heritability estimates were lower than those described in the literature for RFI, ranging from 0.17 to 0.28 [4, 14, 37, 38]. For DMI, the heritability estimates were within the range observed in other Nellore cattle studies, with values ranging from 0.23 to 0.47 [4, 14, 37, 38]. However, the differences in heritability estimates are probably due to the differences between populations (as genetic parameters are population-specific due to differences in allele frequencies), environmental conditions, and the statistical model used to estimate the variance components. Higher genetic responses are expected at EG levels of 1 for RFI and at EG levels of 1 and 9 for DMI due to higher heritability estimates (Fig. 1).

In a study that estimated genetic parameters for feed efficiency traits in crossbred cattle fed growing and finishing diets under successive feeding regimes, Durunna et al. [9] reported higher heritability values for DMI and RFI (0.43 and 0.36, respectively) with the finisher-fed regime than with the grower-fed regime (0.30 and 0.19, respectively). The authors justified these results by the greater additive genetic variation for DMI and RFI in the finisher-fed group than in the grower-fed group. The estimates of the additive genetic variance for DMI increased gradually as environmental conditions improved (0.26 to 0.75), i.e., better environments (assessed based on greater ADG) enhance the differences between animals for this trait. Regarding RFI, the additive genetic variance showed a constant behavior along the EG (0.07 to 0.11; Fig. 1).

The phenotypic variance estimated for RFI and DMI increased across EG levels, ranging from 0.27 to 1.08 and from 0.55 to 1.71, respectively. Thus, a less restricted environment (higher ADG) resulted in the largest phenotypic variability for both traits. However, for RFI, this increase was not due to the greater additive genetic variance, but it reflected the increase in environmental variance, and consequently, the heritability estimates decreased for RFI along the EG. Therefore, the influence due to variance heterogeneity was greater for RFI than for DMI, probably because RFI had a larger influence on environmental variance along the EG.

### Genetic correlation estimates for RFI and DMI across environmental gradients

**Fig. 2:**
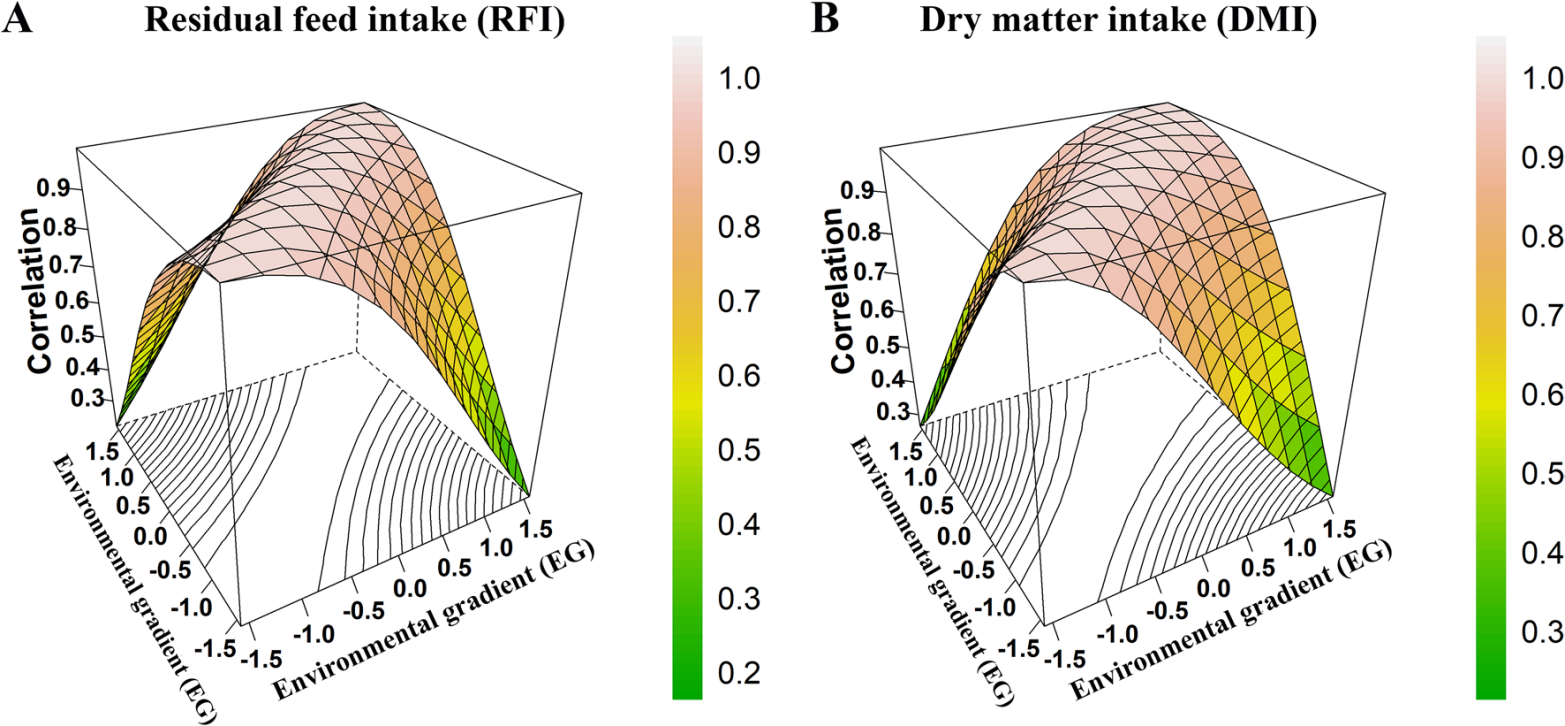
Genetic correlation estimates for residual feed intake (RFI) (**a**) and dry matter intake (DMI) (**b**) across environmental gradients (EG) in Nellore cattle. The colors indicate the magnitude of the genetic correlations

The genetic correlation estimates for the evaluated traits across EG levels ranged from 0.22 to 0.99 (0.81 ± 0.21) for RFI and from 0.26 to 0.99 (0.83 ± 0.19) for DMI, which indicates the presence of G × E interactions (Fig. 2). When the EG levels were more similar, we observed genetic correlation estimates higher than 0.80, and as the EG levels were more divergent, the genetic correlation decreased below 0.80, indicating the occurrence of G × E interactions across EG [7]. The lower genetic correlations between the extreme EG levels represent a significant effect of G × E interactions, potentially leading to reranking of breeding animals due to variation in EBV across EG levels [11, 39]. Genetic correlations close to 0.80 were obtained by Godinho et al. [35] for RFI and average daily feed intake in pigs during the early phase, and below 0.80 for RFI (0.74) in the grower phase with two diets. The authors reported that the genetic progress observed when applying selection under one diet might not be the same as that observed when selecting animals with another diet during these phases due to a reranking of the genotypes. Thus, in pigs, these traits are sensitive to changes in the source of energy nutrients in the diets.

Durunna et al. [9] evaluated G × E interactions for feed efficiency traits in crossbred cattle that were fed growing and finishing diets under successive feeding regimes and reported the existence of G × E interactions for DMI and RFI. Low genetic correlation estimates were observed between DMI (0.63) and RFI (0.39) measures with the two diets, indicating differential performance across environments, i.e., feed efficiency may depend on the diet provided. In this context, G × E models provide a new tool for the evaluation of traits measured in different environments where genetic heterogeneity might exist.

Genetic correlation estimates between the same trait evaluated in different environments have been used to evaluate the degree of the sensitivity of animals to environmental variations for many traits of economic importance in beef cattle [11, 16, 34, 39, 40]. When the genetic correlation between EG levels is below 0.80, the genes that control the additive genetic variance differ between environments or act differently [22]. Thus, the results presented here show evidence of G × E interactions on RFI and DMI, which reflect changes in genetic parameters according to environmental fluctuations, i.e., different ADG in feeding trials influenced the additive genetic variance estimates of DMI and RFI.

### Estimates of the genetic and phenotypic correlations between RFI and DMI across EG

**Fig. 3:**
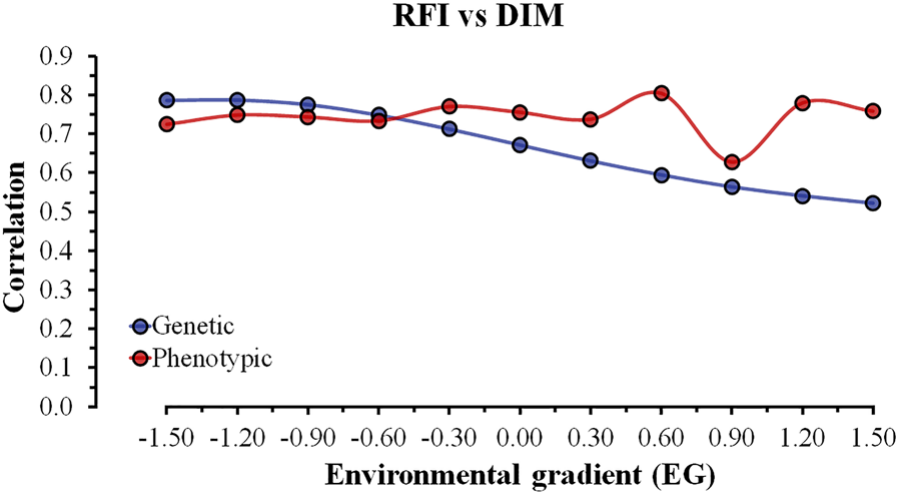
Genetic and phenotypic correlation estimates between residual feed intake (RFI) and dry matter intake (DMI) across environmental gradients (EG) in Nellore cattle

The estimates of genetic and phenotypic correlations between RFI and DMI across EG were positive, ranging from 0.52 to 0.79 and from 0.63 to 0.81, respectively (Fig. 3). These results were expected since RFI is the residual of the regression equation between observed DMI, ADG, and MBW. Previous studies that estimated genetic and phenotypic correlations between DMI and RFI without considering G × E interactions obtained values ranging from 0.51 to 0.85 and from 0.70 to 0.81, respectively [37, 41–44]. This association between RFI and DMI has been widely explored since more efficient animals evaluated for RFI are usually animals that consume less feed, i.e., selecting animals with a negative RFI should lead to a decrease in DMI and consequently to a decrease in the nutritional requirements of the herd without changing the performance of the animals [44, 45]. Although the magnitude of the genetic correlations between RFI and DMI across environments indicates a similar genetic background between these traits, especially in more restricted environments (EG 1 to EG 3), there was a decrease in the magnitude of the correlations as the EG level increased (less restricted environmental groups).

In restricted EG levels (lower ADG), animals showed greater efficiency due to their lower DMI, resulting in lower RFI values. Based on these results, animals with a higher genetic potential for feed efficiency (i.e., lower RFI) tend to have lower DMI. In spite of the nutritional recommendation to provide a diet to attain an ADG of 1 kg/day during the feeding trial [5], differences in diet quality across tests can be expected due to the bromatological variation of the diets. The ability of an animal to use energy from the diet provided is associated with the rumen microbial population, converting the diet energy into body weight gain [46]. Allen [47] and Mertens [48] concluded that physical and physiological factors regulating feed intake change with increasing digestibility or diet quality. Thus, differences in diet quality across tests would affect the regulation of feed intake and consequently feed efficiency.

A reduction in feed efficiency was observed in less restricted EG conditions (Table 4), probably due to the larger residual effect produced by a higher proportion of animals from farms with greater selection emphasis on growth traits, which increases the maintenance requirements and phenotypic mean in the most favorable EG. However, for a better understanding of the genetic mechanisms that are involved in feed efficiency, the use of genomic information could provide a better explanation of the effects of G × E interactions on these traits. In the literature, there are few studies that have estimated the genetic correlations between traits related to feed efficiency, such as RFI and DMI, measured in different environments. Bi-trait RN models are a promising tool for genetic evaluation programs, as they allow the evaluation of the heterogeneity of variances in different environments for traits of economic importance. Therefore, the results presented in this study provide support and information to researchers and breeders to define appropriate selection criteria for specific environments for genetic improvement of feed efficiency-related traits in beef cattle populations that are raised in tropical conditions.

### Genotype-by-environment interactions

**Fig. 4:**
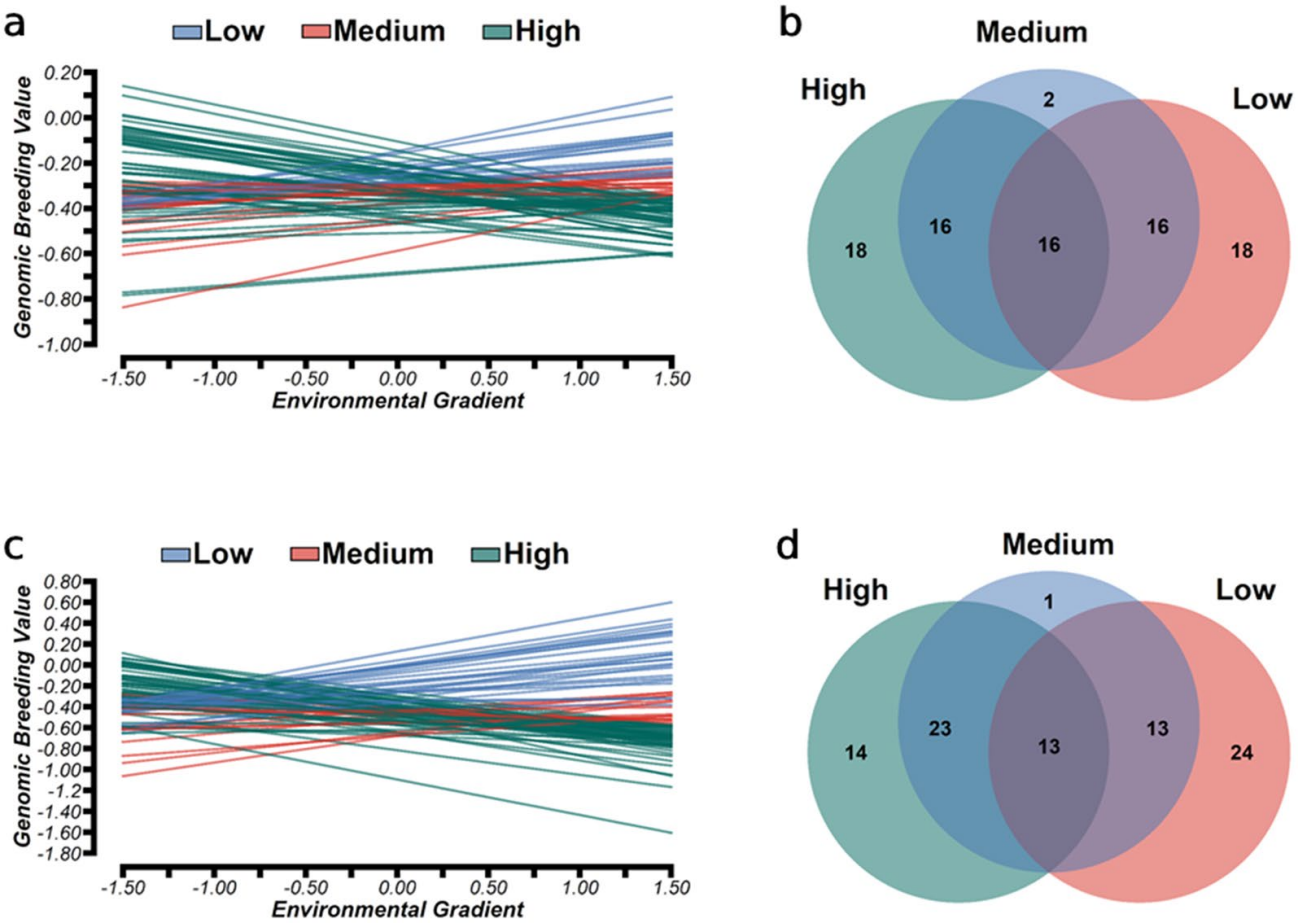
Reaction norms for residual feed intake (RFI) (**a**), and dry matter intake (DMI) (**c**), and the number of matching and specific sires in the environments for RFI (**b**) and DMI (**d**) considering the 50 sires with the highest number of progeny number and top-ranked by EBV for RFI and DMI in the moderate environmental gradient (EG = 0.0)

The RN for 50 sires with the largest number of progeny (average of 86.04, ranging from 27 to 451) that were distributed in at least three EG levels, i.e., low, medium and high EG for RFI and DMI, showed reranking among these sires (Fig. 4a). The effect of G × E interactions on the sensitivity of animals across EG levels, especially between extreme EG levels, was expected due to a genetic correlation lower than 0.80 (Fig. 2). The average EBV for RFI and DMI were, respectively, − 0.027 kg/DM/day and 0.103 kg/DM/day for the low EG level (− 1.5), − 0.053 kg/DM/day and 0.122 kg/DM/day for the medium EG level (0.0), and − 0.085 kg/DM/day and 0.147 kg/DM/day for the high EG level (1.5) (see Additional file 1: Fig. S1). The pattern of the RN reflects the significance of the G × E interactions with the genotypes that have a high plasticity, i.e., a greater sensitivity to environmental changes, being associated with steeper slopes, while the more robust genotypes have flatter slopes. Based on the slope ($f1$) solutions, 43.3% and 42.9% of the animals were classified as highly plastic to environmental changes, and 56.7% and 57.1% as more robust to environmental changes for RFI and DMI, respectively. Among the more robust animals, i.e., ($\left|{f}_{1}\right|<{\sigma }_{f1}$), at a medium EG level, 60% were considered better for both traits and 40% were considered better for one or neither of the traits, while 53% and only 40% were considered better for both traits at a low and high EG level, respectively.

The sire EBV for RFI and DMI across EG levels followed similar trends (Fig. 4a). However, comparing the EBV of the top 15 sires for both traits (see Additional file 2: Tables S1 and S2), larger differences in EBV for DMI were observed between EG levels (see Additional file 2: Table S2), as indicated by the lower genetic correlation between extreme EG levels (Fig. 2b). Animal sensitivity to environmental variations plays a role in the phenotypic mean and the trait’s genetic variance under different environmental conditions [49]. RFI and DMI showed changes in the phenotypic mean (Table 4) and genetic variance (Fig. 1) according to EG level, and it is important to carefully evaluate animal selection for feed efficiency traits as the environment becomes more divergent (e.g., greater diet variability). Spearman correlation and selection coincidence were estimated by selecting the 50 sires with at least five progenies raised at low, medium, and high EG levels and with the highest EBV to visualize the effect of G × E interactions (Table 4). When the top 50 sires that were ranked based on the EBV of animals with at least five progenies raised at low, medium, and high EG levels were considered, the Spearman’s correlation values were highest between the medium EG level and either the low or high EG levels for both traits (Table 5). These results indicate that the selection of animals for feed efficiency based on data from feeding trials with an expected ADG around 1 kg/day had less impact on sires’ rank across environments compared to selection based on data from extreme EG levels.

**Table 5:** Spearman's rank correlation and selection coincidence of top 50 sires for residual feed intake (RFI) and dry matter intake (DMI) with at least five progenies in three different environmental gradients

Scenarios^a^	Spearman's correlation	Selection coincidence^b^	
Residual feed intake (RFI)			
Medium EG vs low EG	0.85	73.3%	
Medium EG vs high EG	0.89	80.0%	
Low EG vs high EG	0.61	53.3%	
Dry matter intake (DMI)			
Medium EG vs low EG	0.83	66.7%	
Medium EG vs high EG	0.90	70.0%	
Low EG vs high EG	0.57	40.0%	
Number of sires and progenies under different environmental gradients (EG)	Low EG	Medium EG	High EG
Number of sires with progeny	45	48	26
Number of average progeny/sire	28	58	9
Total number of progenies	1273	2792	237

^a^Medium environmental gradient (EG) is the comparison criterion for selection coincidence

^b^Represents the percentage of sires in common between environments gradients evaluated

**Fig. 5:**
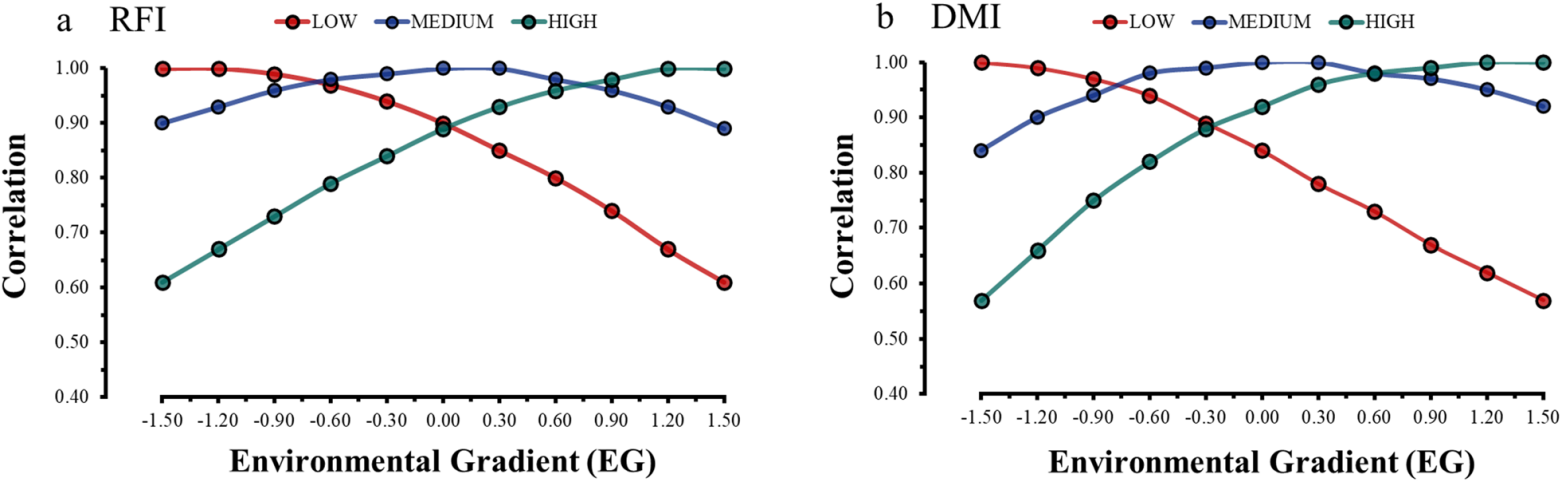
Pearson’s correlation between the sire estimated breeding values (EBV) obtained in the low (red), moderate (blue), and high (green) environmental gradients (EG) across EG levels for residual feed intake (RFI) (**a**) and dry matter intake (DMI) (**b**)

The selection coincidence of the sires that were ranked based on the highest EBV and the largest number of progenies between the medium and low EG levels was 73.33% for RFI and 66.67% for DMI, and between the medium and high EG levels it was 80% for RFI and 70% for DMI (Table 5). These results indicate that most of the bulls with a superior genetic potential for feed efficiency based on the performance of their progeny maintain this potential for feed efficiency at extreme EG levels. However, we compared the selection of animals performed under extreme EG levels (low EG vs. high EG), and found lower Spearman correlations, 0.61 and 0.55, and lower selection coincidence percentages among the bulls, being 53.3% and 40.0%, for RFI and DMI (Table 5), respectively, which indicate a clear reranking of sires across extreme EG. These results indicate that selection decisions for feed efficiency-related traits based on EBV from more restricted environments would affect the ranking of sires in less restricted environments (and vice versa). Spearman’s correlations for the EBV of the 50 sires with the largest progeny number between low and high EG levels were equal to 0.61 for RFI (Fig. 5a) and 0.57 for DMI (Fig. 5b). These results indicate that these traits are influenced by G × E interactions when a smaller number of animals are shared among EG levels (Fig. 4). We observed that among the top 50 sires, only 16 for RFI (Fig. 4a) and 13 for DMI (Fig. 4b) were shared among the low, medium, and high EG levels.

The Spearman’s correlations when comparing sire EBV for RFI and DMI at the medium EG level with those at the other EG levels ranged from 0.85 to 1.00 (Fig. 5a) and from 0.83 to 1.00 (Fig. 5b), respectively, which indicates a small change in EBV ranking. Thus, on the one hand, the top sires selected at the medium EG level are expected to maintain their genetic superiority across EG levels. On the other hand, when the sires’ ranks were compared between extreme environmental conditions (i.e., low EG or high EG level), greater reclassification of sires was expected (Fig. 5). From a practical point of view, when animals are selected for RFI and DMI under feeding trials with ADG around 1 kg/day, small changes in animal performance, or in reranking of top sires for RFI and DMI are expected. The presence or evidence of G × E interactions for RFI and DMI caused small changes in the sires’ EBV when selection was applied at a medium EG level, and major reranking of the top sires in extreme environments. Therefore, it is crucial to follow the recommendations proposed by Mendes et al. [5], i.e. to adequately measure or collect RFI and DMI records in feed efficiency trials under different conditions when performing national genetic evaluations for these traits.

## Conclusions

Our results show clear evidence of genotype-by-environment interactions on feed efficiency indicator traits in Nellore cattle. The breeding values for residual feed intake and dry matter intake were sensitive to environmental changes. This interaction was particularly clear in more divergent environments, e.g. when the variance in average live weight gain was substantial during the feeding trials. Furthermore, as the environmental conditions were less restricted (better environments), the expected correlated response in residual feed intake based on selection for dry matter intake is expected to decrease (and vice versa) since the genetic association between these traits was smaller in less restricted (better) environments. From a practical point of view, when animals are selected for residual feed intake and dry matter intake under feeding trials that allow an average daily gain of approximately 1 kg/day (i.e., from 0.9 to 1.4 kg/day), a slight change in animal performance is expected. However, when animals are selected for both traits in feeding trials with an ADG that is far from the average value, an increase in reranking is observed, which may be caused by the difference in nutritional levels masking the genetic potential and biasing the genetic evaluations for feed efficiency in progeny that are fed for a different ADG.

### Supplementary Information

**Additional file 1: Figure S1.** Boxplot of the estimated breeding values (EBV) of 50 sires with the largest progeny number for residual feed intake (RFI) and dry matter intake (DMI) in low, medium, and high environmental gradient (EG) levels.

**Additional file 2: Table S1.** Correspondence of selection of the top 15 Nellore sires with the largest progeny number, ranked by EBV for residual feed intake (RFI) in the medium, low and high environmental gradients (EG). Correspondence of selection of the top 15 Nellore sires with the highest progeny number, ranked by EBV for dry matter intake (DMI) in the medium, low and high environmental gradients (EG).

## Data Availability

Phenotypic and genotypic information is available for academic use from the authors upon reasonable request (Dr. João Carlos G. Giffoni Filho, President of ANCP email: presidencia@ancp.org.br).
