# Supplementary material for: Genotype-by-environment interactions for feed efficiency traits in Nellore cattle based on bi-trait reaction norm models
Source: Genet Sel Evol. 2023 Dec 14;55:93. doi: 10.1186/s12711-023-00867-2 (PMC10722809; doi:10.1186/s12711-023-00867-2)
Supplement: Supplementary file 1 — Additional file 1: Figure S1. Boxplot of the estimated breeding values (EBV) of 50 sires with the largest progeny number for residual feed intake (RFI) and dry matter intake (DMI) in low, medium, and high environmental gradient (EG) levels. [file 12711_2023_867_MOESM1_ESM.docx]

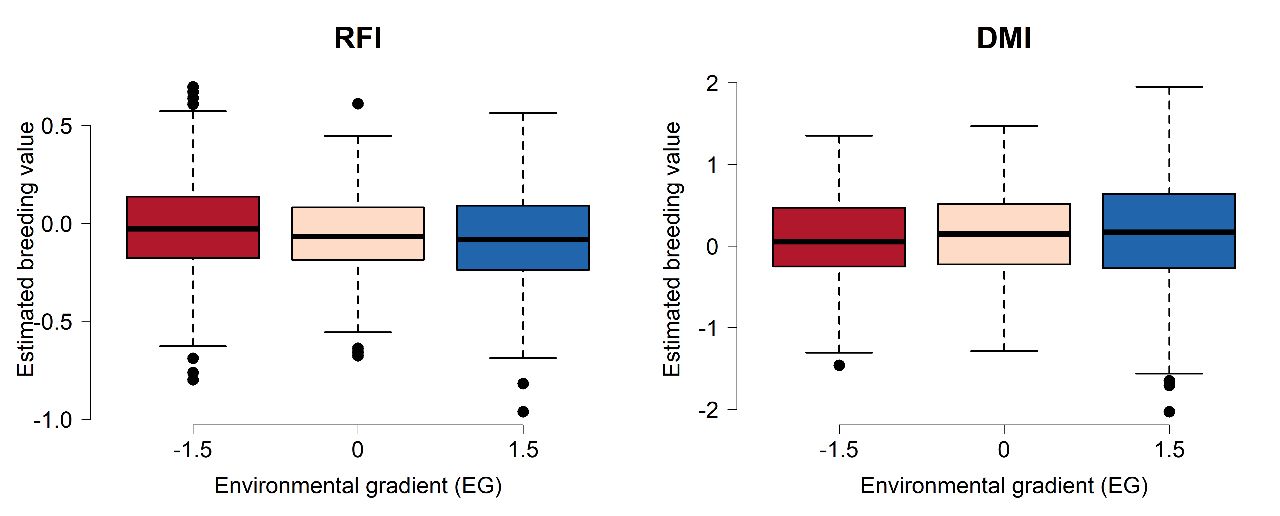


**Figure S1.** Boxplot of 50 sires’ estimated breeding values (EBV) with the highest number of progeny number for residual feed intake (RFI) and dry matter intake (DMI) in low, medium, and high environmental gradient (EG) levels.
