# Supplementary material for: Genotype-by-environment interactions for feed efficiency traits in Nellore cattle based on bi-trait reaction norm models
Source: Genet Sel Evol. 2023 Dec 14;55:93. doi: 10.1186/s12711-023-00867-2 (PMC10722809; doi:10.1186/s12711-023-00867-2)
Supplement: Supplementary file 2 — Additional file 2: Table S1. Correspondence of selection of the top 15 Nellore sires with the largest progeny number, ranked by EBV for residual feed intake (RFI) in the medium, low and high environmental gradients (EG). Correspondence of selection of the top 15 Nellore sires with the highest progeny number, ranked by EBV for dry matter intake (DMI) in the medium, low and high environmental gradients (EG). [file 12711_2023_867_MOESM2_ESM.docx]

**Table S1.** Correspondence of selection of the top 15 Nellore sires with the highest number of progeny, ranked based on their estimated breeding value (EBV) for residual feed intake (RFI) in low, medium, and high environmental gradients (EG).

| Ranking EG Medium | EBV EG Medium | Ranking EG Low | EBV EG Low | Ranking EG High | EBV EG High | Total number of progeny |
| --- | --- | --- | --- | --- | --- | --- |
| 1 | -0.643 | 1 | -0.684 | 3 | -0.591 | 32 |
| 2 | -0.534 | 3 | -0.455 | 2 | -0.632 | 58 |
| 3 | -0.515 | 13 | -0.191 | 1 | -0.921 | 149 |
| 4 | -0.474 | 6 | -0.403 | 4 | -0.563 | 52 |
| 5 | -0.454 | 7 | -0.401 | 6 | -0.521 | 61 |
| 6 | -0.402 | 2 | -0.462 | 12 | -0.328 | 85 |
| 7 | -0.363 | 5 | -0.439 | 13 | -0.267 | 50 |
| 8 | -0.329 | 9 | -0.324 | 10 | -0.335 | 40 |
| 9 | -0.328 | 15 | -0.152 | 5 | -0.548 | 37 |
| 10 | -0.293 | 4 | -0.448 | 22 | -0.100 | 112 |
| 11 | -0.265 | 18 | -0.090 | 7 | -0.484 | 149 |
| 12 | -0.246 | 14 | -0.168 | 9 | -0.344 | 39 |
| 13 | -0.237 | 11 | -0.274 | 17 | -0.191 | 27 |
| 14 | -0.234 | 17 | -0.105 | 8 | -0.395 | 451 |
| 15 | -0.190 | 12 | -0.271 | 24 | -0.087 | 31 |

* Ranking medium EG is the comparison criterion.

**Table S2.** Correspondence of selection of the top 15 Nellore sires with the highest progeny number, classified by EBV for dry matter intake (DMI) in the medium, low and high environmental gradients (EG).

| Ranking EG Medium | | EBV EG Medium | | Ranking EG Low | | EBV EG Low | | Ranking EG High | | EBV EG High | | Total number of progeny | |
| --- | --- | --- | --- | --- | --- | --- | --- | --- | --- | --- | --- | --- | --- |
| 1 | -1.138 | | 1 | | -1.424 | | 6 | | -0.779 | | 32 | |  |
| 2 | -0.942 | | 2 | | -0.700 | | 3 | | -1.244 | | 149 | |  |
| 3 | -0.777 | | 17 | | -0.102 | | 1 | | -1.622 | | 31 | |  |
| 4 | -0.728 | | 10 | | -0.296 | | 2 | | -1.267 | | 30 | |  |
| 5 | -0.564 | | 7 | | -0.420 | | 7 | | -0.744 | | 89 | |  |
| 6 | -0.537 | | 6 | | -0.427 | | 8 | | -0.674 | | 39 | |  |
| 7 | -0.491 | | 14 | | -0.164 | | 4 | | -0.900 | | 112 | |  |
| 8 | -0.474 | | 15 | | -0.152 | | 5 | | -0.877 | | 58 | |  |
| 9 | -0.456 | | 4 | | -0.679 | | 14 | | -0.177 | | 52 | |  |
| 10 | -0.398 | | 3 | | -0.685 | | 22 | | -0.040 | | 85 | |  |
| 11 | -0.367 | | 11 | | -0.287 | | 11 | | -0.466 | | 27 | |  |
| 12 | -0.356 | | 5 | | -0.502 | | 15 | | -0.173 | | 37 | |  |
| 13 | -0.325 | | 12 | | -0.272 | | 12 | | -0.391 | | 48 | |  |
| 14 | -0.300 | | 9 | | -0.344 | | 13 | | -0.245 | | 31 | |  |
| 15 | -1.138 | | 19 | | -1.424 | | 10 | | -0.779 | | 50 | |  |

* Ranking medium EG is the comparison criterion.
